# Aberrant Mannosylated and Highly Fucosylated Glycoepitopes of Prostatic Acid Phosphatase as Potential Ligands for Dendritic-Cell Specific ICAM-Grabbing Nonintegrin (DC-SIGN) in Human Seminal Plasma—A Step towards Explaining Idiopathic Infertility

**DOI:** 10.3390/biom14010058

**Published:** 2023-12-31

**Authors:** Anna Kałuża, Katarzyna Trzęsicka, Damian Drzyzga, Mirosława Ferens-Sieczkowska

**Affiliations:** 1 Department of Biochemistry and Immunochemistry, Division of Chemistry and Immunochemistry, Wroclaw Medical University, M. Skłodowskiej-Curie 48/50, 50-369 Wroclaw, Poland; miroslawa.ferens-sieczkowska@umw.edu.pl; 2 INVICTA, Research and Development Center, Polna 64, 81-740 Sopot, Poland; katarzyna.trzesicka@invicta.pl (K.T.); damian.drzyzga@invicta.pl (D.D.)

**Keywords:** prostatic acid phosphatase, DC-SIGN, dendritic-cell specific ICAM-grabbing nonintegrin, seminal plasma, idiopathic infertility

## Abstract

Semen prostatic acid phosphatase (PAP) has been proposed as an endogenous ligand for dendritic cell-specific ICAM-3-grabbing nonintegrin (DC-SIGN), which plays a critical immuno-modulating role in maintaining homeostasis in the female reproductive tracts. In the current study, we assumed that semen PAP bears a set of fucosylated and mannosylated glycans, which may mediate the efficient binding of PAP to DC-SIGN. To investigate this hypothesis, we developed ELISA assays using *Galanthus nivalis* and *Lotus tetragonolobus* lectins capable of binding mannose-containing glycans or LewisX and LewisY motifs, respectively. In our assay with *Galanthus nivalis,* we detected that the relative reactivity of PAP mannose-presenting glycans in the normozoospermic idiopathic group was significantly higher than in the asthenozoospermic, oligozoospermic and oligoasthenozoospermic groups. Simultaneously, we observed slight differences in the relative reactivities of PAP glycans with *Lotus tetragonolobus* lectin among groups of patients with abnormal semen parameters. Subsequently, we examined whether DC-SIGN interacts with seminal plasma PAP glycans, and we detected a significantly higher relative reactivity in the normozoospermic group compared to the oligozoospermic group. Finally, we concluded that the significantly aberrant abundance of mannosylated functional groups of PAP among patients with semen disorders can suggest that PAP may thereby be engaged in modulating the immune response and promoting a tolerogenic response to male antigens in the female reproductive system.

## 1. Introduction

Human prostatic acid phosphatase (PAP or ACPP) is a 100 kDa secreted glycoprotein enzyme consisting of the two subunits (50 kDa) synthesized in columnar prostate epithelial cells [1]. PAP is one of the major proteins in seminal fluid (0.3–1.0 mg/mL). This can suggest a significant role of the protein in fertility; for example, its participation in increasing sperm motility has been indicated [2]. PAP, as a non-specific phosphomonoesterase, catalyzes the hydrolysis of different phosphate esters, including phosphocholine and phosphocreatine, which are compounds known to serve as energy reserves in the seminal fluid [2]. One of the previous studies has demonstrated that the PAP level inversely correlates with sperm concentration [1,3]. Moreover, another group found that the highest phosphatase activity was detected in azoospermic men, and sperm concentrations tended to return to correct values when phosphatase activity decreased [1,4]. Additionally, Curi et al. proposed a direct relationship between PAP concentration and sperm motility. They proved that PAP concentration decreased in asthenozoospermic patients with impaired sperm motility [5].

PAP produced in prostate epithelial cells exists in two forms: intracellularly as the cellular form (cPAP) and as the secreted form (sPAP) in seminal fluid. Both forms exhibit differences in biochemical characteristics such as isoelectric point values, hydrophobicity and glycosylation patterns [1,6,7,8]. PAP contains three glycosylation sites at Asn-62, Asn-188, and Asn-301. The recent analysis has revealed that sialylated complex-type glycans occupy the site at Asn-62, with bi- and triantennary structures present. The oligosaccharides attached to Asn-188 are most likely the tetrantennary complex sialylated and fucosylated structures, whereas the high-mannose glycoforms are primarily present at the Asn-301 site, with Man6 and Man7 glycans observed for this site [9]. The glycosylation and six cysteine residues forming two disulfide bonds support the stability of human PAP protein, glycosylation sites are indeed conserved in all mammalian PAPs [1,8].

Mass spectrometry approaches have confirmed the occurrence of *N*-glycans bearing antennae terminated with multiple LewisX and LewisY epitopes on glycoproteins present in human seminal plasma and in purified human spermatozoa [10,11,12,13,14]. Interestingly, prostatic acid phosphatase has been proposed as endogenous ligand for DC-SIGN (dendritic cell-specific ICAM-3-grabbing nonintegrin; CD209) along with several other seminal plasma glycoproteins, such as clusterin, galectin-3 binding glycoprotein, and protein C inhibitor, the mentioned ligands could play an important role in maintaining immune homeostasis in the male urogenital tract and the vagina after coitus [10,15]. DC-SIGN is a type II transmembrane C-type lectin bearing a conserved carbohydrate recognition domain (CRD), which recognizes at least two classes of oligosaccharides: high mannose type *N*-glycans and several Lewis type carbohydrate sequences, namely LewisA, LewisB, LewisX, and LewisY [16]. van Liempt et al. showed that DC-SIGN-Fc chimera protein interacts with α1–3 and α1–4 fucosylated tri- and tetrasaccharides; thus, LewisB exhibits the highest relative binding, followed by LewisY and LewisA. Additionally, DC-SIGN-Fc binds to *N*-linked oligomannose with the highest affinity for *N*-glycan structures carrying 5–9 mannose residues and the stated decreasing binding apparent affinity as the number of mannoses decreases [16].

In the present study, the concentration of PAP was quantified using a highly sensitive and specific enzyme-linked immunosorbent assay (ELISA) in the seminal fluid of fertile and infertile men. Moreover, we report here that semen prostatic acid phosphatase bears a set of fucosylated and mannosylated glycans that may mediate the efficient binding of semen PAP to DC-SIGN. To investigate this hypothesis, we developed ELISA assays based on the ability of the two different lectins, *Galanthus nivalis* and *Lotus tetragonolobus,* to bind mannose-containing or terminal fucose (LewisX and LewisY) motifs, which are excellent DC-SIGN ligands. Subsequently, in a preliminary experiment with the direct ELISA format, which is the simplest and quickest to perform, we examined whether DC-SIGN interacts with seminal plasma PAP and if noticeable differences were present in this interaction according to the investigated groups of patients.

## 2. Materials and Methods

### 2.1. Seminal Plasma Samples

Seminal plasma (SP) samples were obtained from 22–49-year-old men attended with partners to INVICTA Fertility Clinic, Wrocław. Semen samples were obtained through masturbation after 2–3 days of sexual abstinence and liquefied within one hour at 37 °C. After standard semen analysis, spermatozoa were gently centrifuged at 400× *g* for 10 min at room temperature. A spare fraction of seminal plasma provided the material for our investigations. Standard semen examination was performed for all the patients (*n* = 115) and control subjects (C; *n* = 20) according to World Health Organization directives (WHO 2010). Based on this testing the samples coming from infertile men were divided into the following groups: asthenozoospermic (A; *n* = 28; total sperm motility < 40%), oligozoospermic (O; *n* = 20; sperm count < 15 × 10^6^/mL), oligoasthenozoospermic (OA; *n* = 23; sperm count < 15 × 10^6^/mL and total sperm motility < 40%), teratozoospermic (T; *n* = 19; abnormal sperm phenotypes), and normozoospermic (N; *n* = 25; semen parameters within WHO normal range). The control group consisted of seminal plasma samples from healthy volunteers who had fathered a child within recent years; all the control subjects were normozoospermic. All the participants provided written informed consent, and the study was approved by the Wrocław Medical University Bioethics Council (approval number KB-535/2019).

### 2.2. Quantification of Prostatic Acid Phosphatase

Seminal plasma prostatic acid phosphatase concentration was determined with a Human Prostatic Acid Phosphatase/ACPP kit (R&D Systems, Minneapolis, MN, USA, catalog No. DY6240-05) in accordance with the enclosed manufacturer’s instruction, and appropriate modifications allowing determination of the protein in seminal plasma samples. Briefly, a 96-well ELISA microtiter plate (Nunc International, Naperville, IL, USA) was coated with mouse anti-human PAP mAb (R&D, 0.5 μg/mL) antibodies diluted in phosphate-buffered saline (PBS) and incubated overnight at room temperature. After the coating solution was removed, the plate was washed five times with PBST (0.05% Tween20 in PBS). The plate was then blocked with 1% BSA in PBS for 2 h at 37 °C. One hundred microliters of each standard from 0.125–4.0 ng/mL in PBS and a 1.000.000-fold or 500.000-fold diluted seminal plasma sample were applied to a plate well and incubated for 1 h at 37 °C. Sheep anti-human PAP biotinylated antibodies (200 ng/mL) were used as a detection antibody and incubated for 2 h at 37 °C. After the next extensive washing of the plate, one hundred microliters of streptavidin-horseradish peroxidase conjugate solution (Streptavidin-HRP), diluted 1:40 in the 0.1% BSA in PBS, was applied to each well. After 20 min of incubation at 37 °C and another extensive washing, 3,3′-5,5′-tetramethylbenzidine (TMB, Sigma–Aldrich, St. Louis, MO, USA) substrate solution was added to each well for 5 min, followed by 1 M H_2_SO_4_ stop solution. The absorbance values were read on a Synergy LX Multi-Mode Reader (BioTek Instruments, Inc., Winooski, VT, USA) at a wavelength of 450 nm. All seminal plasma samples were assayed in duplicate; the mean value of the two replicates was used for statistical analysis. The results were expressed in the arbitrary units (AU) after subtracting the absorbance of the blank samples. The intra-assay and inter-assay coefficients of variations (CV%) for seminal plasma PAP concentration were calculated as CV% 2.4 and CV% 6.2, respectively.

### 2.3. Mild Oxidation of the Glycans on Capture Antibodies

Since antibodies are glycosylated themselves, lectins applied as detection agents can bind to the *N*-glycans in Fc region of antibodies immobilized on the plate, which may lead to cross-reactivity interfering with the specific detection of glycans on the protein captured by the antibodies. The effective approach to prevent this interference is to chemically modify the glycans on the capture antibodies through the gentle oxidation of *cis*-hydroxyl groups on the used antibodies to convert them into aldehyde groups, which are later reacted with a hydrazidemaleimide cross-linking reagent (MPBH), followed by further reacting with a cysteine-glycine (Cys-Gly) dipeptide [17,18].

The oxidation procedure was carried out according to the Thermo Scientific manual for the MPBH product. Fresh 20 mM of MPBH (Thermo Fisher Scientific, Waltham, MA, USA) was prepared by dissolving the appropriate amount of the compound in dimethylformamide (DMF, Sigma–Aldrich, St. Louis, MO, USA). Next, a five-fold molar excess of crosslinker was added to sulfhydryl-containing dipeptides (10 mM Cys-Gly, Sigma–Aldrich, St. Louis, MO, USA). The reaction mixture was incubated for 2 h at room temperature. Then, desalting columns equilibrated with coupling buffer pH 7.4 were used to remove reagent excess and for buffer exchange. Subsequently, 100 mM sodium *meta*-periodate (NaIO_4_, Thermo Fisher Scientific, Waltham, MA, USA) in oxidation buffer was prepared, and the solution was kept on ice and protected from light.

Afterward, 1 mL of cold fresh sodium *meta*-periodate was added to 1 mL of the antibodies solution and mixed well. The oxidation reaction proceeded in the dark for 30 min on ice. In the final step, a desalting column equilibrated with crosslinked buffer pH 7.4 was used to remove excess periodate and exchange the buffer. The final conjugation of the crosslinker-modified sulfhydryl dipeptides and the oxidized antibodies was performed by combining them in appropriate proportions (1:1). The reaction mixture was incubated for 2 h at room temperature. The effectiveness of the method was checked by comparing the absorbance value in the reaction with the tested lectins before and after the antibody oxidation reaction, stating a reduced absorbance value (A ≤ 0.1) in relation to the initial sample.

### 2.4. Detection of Mannosylated and Fucosylated Glycoepitopes of Prostatic acid Phosphatase

An ELISA plate (Nunc International, Naperville, IL, USA) was coated with 100 μL of mouse anti-human prostatic acid phosphatase monoclonal antibodies (R&D Systems, Minneapolis, MN, USA, catalog No. DY6240-05), prepared as described above. The antibodies were applied in a concentration of 1 μg/mL and incubated overnight at room temperature. Afterward, the plate was washed five times with 10 mM PBS, pH 7.4. Then, free binding sites of the plate wells were blocked with 1% BSA in 10 mM PBS for 1.5 h at 37 °C. The samples were diluted in 10 mM PBS, pH 7.4 buffer to obtain a PAP concentration 2 μg/100 μL per well. Then, each seminal plasma sample was applied to a well plate in duplicate and incubated for 2 h at 37 °C with gentle shaking. In the next step, after washing with 10 mM PBS containing 0.05% Tween20, the biotinylated *Galanthus nivalis* lectin (GNL, Vector Labs, Peterborough, UK, 4 μg/mL) or *Lotus tetragonolobus* lectin (LTL, Vector Labs, Peterborough, UK, 2 μg/mL) was added, and incubated for 1 h at 37 °C. The presence of mannosylated and fucosylated glycoepitopes was revealed using ExtrAvidin-AP (ExtrAvidin-alkaline phosphatase labeled, Sigma–Aldrich, St. Louis, MO, USA), diluted 1:5000 and incubated for 30 min at 37 °C. Next, disodium *para*-nitrophenyl phosphate substrate (*p*-NPP, Thermo Fisher Scientific, Waltham, MA, USA, 1.0 mg/mL) was added, and after 20 min, the reaction was stopped with 1 M NaOH. The absorbance values were read on a Synergy LX Multi-Mode Reader (BioTek Instruments, Inc., Winooski, VT, USA) at a wavelength of 405 nm with a reference filter of 630 nm. The final values were marked in the arbitrary unit (AU) with a subtraction of the absorbance of the blank sample. Glycophorin, protein-bearing sialylated glycans, was used as a negative control to confirm the specificity of *Galanthus nivalis* and *Lotus tetragonolobus* lectins. The intra-assay and inter-assay coefficients of variations for related reactivity of PAP glycans with mentioned lectins in seminal plasma were calculated as CV% 1.7 and CV% 7.3 for GNL while CV% 2.8 and CV% 8.6 for LTL.

### 2.5. DC-SIGN Interaction with PAP

Nunc Maxisorp ELISA plate was coated directly with DC-SIGN protein (Human CD209, LSBio, Lynnwood, WA, USA) in 0.5 μg/mL concentration for 2 h at 37 °C. After washing, the plate was blocked with 10 mM PBS buffer containing 1% BSA for 90 min at 37 °C. For PAP binding, the plate was washed in 10 mM PBS containing 0.05% Tween20 and incubated with seminal plasma samples diluted in 20 mM Tris-HCl, 150 mM NaCl containing 1 mM Ca^2+^, 1 mM Mg^2+^ buffer. An adequate amount of PAP was established in the preliminary experiment as 0.05 μg/100 μL per well. Afterward, each seminal plasma sample was applied to a plate well in duplicate and incubated for one hour at 37 °C with gentle shaking. For PAP determination, the plate was incubated for one hour at 37 °C with biotinylated anti-PAP antibodies (R&D Systems, Minneapolis, MN, USA, 60 ng/mL). After washing, bound PAP was detected by Streptavidin-HRP solution (R&D Systems, Minneapolis, MN, USA, 1:100 diluted). The reaction was visualized with TMB substrate (Sigma–Aldrich, St. Louis, MO, USA, 100 μg/mL), and the reaction was stopped after 10 min with 12.5% H_2_SO_4_. Absorbance values were read on a Synergy LX Multi-Mode Reader (BioTek Instruments, Inc., Winooski, VT, USA) at a wavelength of 450 nm with a reference filter of 630 nm. As previously stated, the final values were shown in the arbitrary unit (AU) with a subtraction of the absorbance of the blank sample. Blank, standard and control samples in duplicates were run alongside every assay to verify that the assay was well-established and repeatable. To evaluate the interaction between DC-SIGN and mannosylated or fucosylated structures on PAP, we performed a competitive ELISA assay, using mannose or fucose as competing ligands separately, which confirms that mannose and fucose glycoepitopes are involved in the interaction of PAP with DC-SIGN. The intra-assay and inter-assay CV% for reactivity of PAP glycoepitopes with DC-SIGN in seminal plasma were calculated as CV% 3.1 and CV% 9.8, respectively.

### 2.6. Statistical Analysis

Statistical analysis was conducted with Statistica 13.3 (StatSoft, Inc., Tulsa, OK, USA). The normality of the distribution of all analyzed parameters was tested with the Shapiro–Wilk test. Prostatic acid phosphatase concentration and relative reactivities with lectins were presented as mean ± SD and illustrated in the box-whisker graphs as the median and the 25–75th percentiles. The Mann–Whitney U-test was used to compare PAP concentration and relative reactivity with studied lectins between the control group (C) and each patient’s group (N, A, O, OA, and T), as well as to calculate a statistical significance of the detected difference. The *p*-values less than 0.05 were considered statistically significant. Additionally, the associations between measured relative reactivities of PAP glycoepitopes with GNL or LTL lectin and the relative reactivity of PAP glycans with DC-SIGN were estimated according to Spearman’s rank correlation for all examined patients. Scatter plots representing the relationship between DC-SIGN reactivity and relative reactivity of PAP glycans with *Galanthus nivalis* or *Lotus tetragonolobus* lectin were performed with the Minitab Statistical Software Version 21.1.0 (Minitab Inc., Pennsylvania State College, PA, USA).

## 3. Results

### 3.1. Concentration of Prostatic Acid Phosphatase

Prostatic acid phosphatase was quantified by ELISA assay in seminal plasma samples for five patient groups (*n* = 115) and control group (*n* = 20) (Table 1).

**Figure 1:**
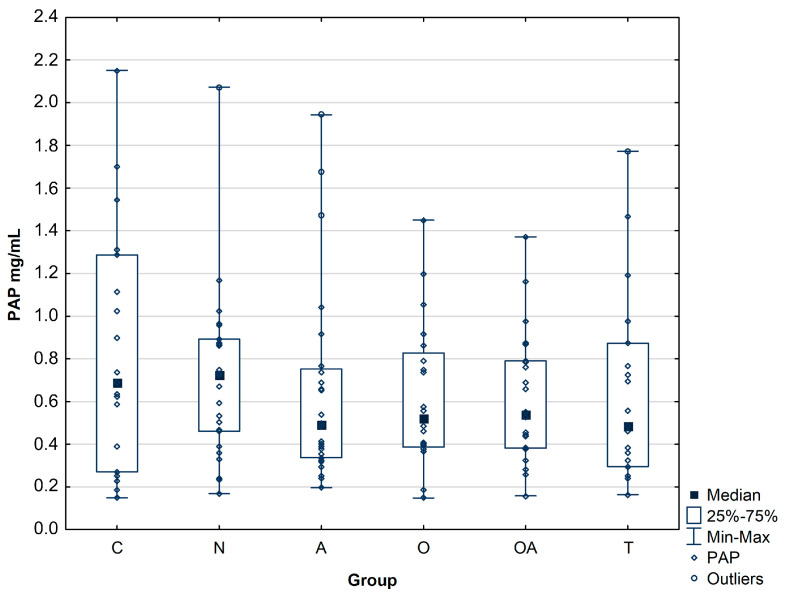
Concentration of prostatic acid phosphatase (PAP) in seminal plasma. Investigated groups: C—control group, N—normozoosperic group, A—asthenozoospermic group, O—oligozoospermic group, OA—oligoasthenozoospermic group, T—teratozoospermic group.

In all the infertile men groups, the concentration of PAP was slightly decreased compared to the controls, although the difference did not reach statistical significance (Figure 1). The mean concentration of PAP was at a similar level for all patient groups, namely 0.71 ± 0.40 mg/mL for normozoospermic, 0.66 ± 0.46 mg/mL for asthenozoospermic, 0.61 ± 0.35 mg/mL for oligozoospermic, 0.61 ± 0.31 mg/mL for oligoasthenozoospermic, 0.65 ± 0.44 mg/mL for theratozoospermic group, while the control group reached a slightly higher value 0.83 ± 0.57 mg/mL (Table 2, Figure 1).

### 3.2. Detection of Mannose and High-Fucose Type Glycoepitopes in Prostatic Acid Phosphatase

To detect mannosylated and fucosylated glycoepitopes of prostatic acid phosphatase, we performed two ELISA tests based on different specificities of the selected lectins: *Galanthus nivalis* or *Lotus tetragonolobus*, which can bind to mannose or antennary fucose present in LewisX and LewisY antigens, respectively.

**Figure 2:**
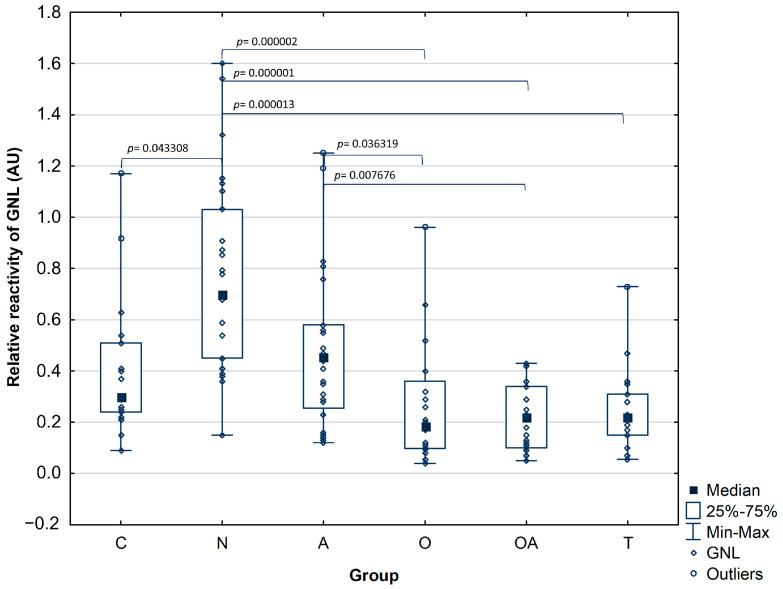
Relative reactivity of prostatic acid phosphatase glycans with mannose-specific lectin GNL-*Galanthus nivalis*. Investigated groups: C—control group, N—normozoosperic group, A—asthenozoospermic group, O—oligozoospermic group, OA—oligoasthenozoospermic group, T—teratozoospermic group.

Relative reactivity of PAP glycans with GNL in seminal plasma of normozoospermic infertile group (median value: 0.78 ± 0.38 AU) was significantly higher in comparison to the fertile control (median value: 0.41 ± 0.27 AU; *p*^C^ = 0.043308) as well as to the infertile subjects with impaired semen parameters: oligozoospermic (median value: 0.26 ± 0.23 AU; *p*^O^ = 0.000002), oligoasthenozoospermic (median value: 0.21 ± 0.12 AU; *p*^OA^ = 0.000001) and theratozoospermic group (median value: 0.25 ± 0.17 AU; *p*^T^ = 0.000013). Additionally, the relative reactivity of seminal plasma PAP glycans with GNL was significantly higher in the asthenozoospermic group (median value: 0.48 ± 0.30 AU) compared to the oligozoospermic and oligoasthenozoospermic group with significance of *p*^O^ = 0.036319 and *p*^OA^ = 0.007676, respectively (Table 3, Figure 2).

**Figure 3:**
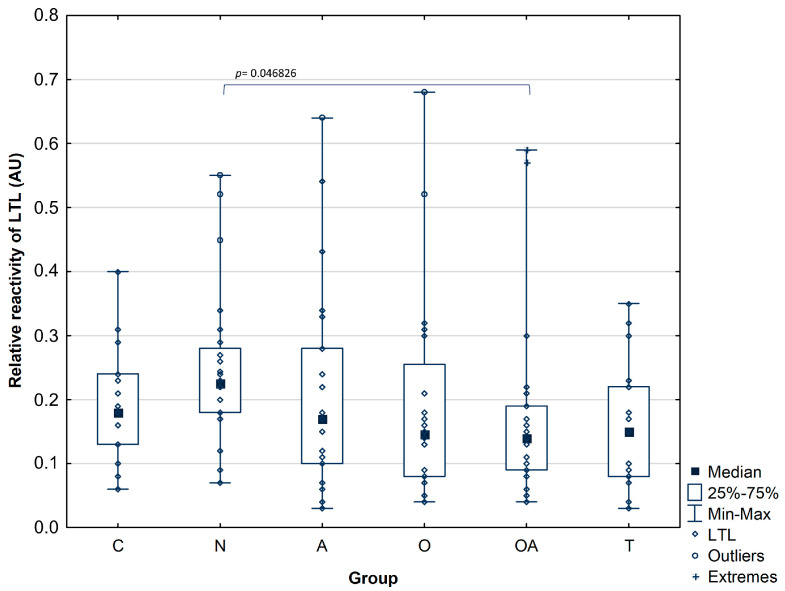
Relative reactivity of prostatic acid phosphatase glycans with fucose-specific lectin LTL-*Lotus tetragonolobus* lectin. Investigated groups: C—control group, N—normozoosperic group, A—asthenozoospermic group, O—oligozoospermic group, OA—oligoasthenozoospermic group, T—teratozoospermic group. Details on statistical significance are provided in Table 3.

When investigating the reactivity of LTL with PAP glycans in the seminal plasma of the oligoasthenozoospermic group (median value: 0.17 ± 0.15 AU; *p*^OA^ = 0.046826), we found a significantly lower LewisX and LewisY motifs expression in comparison to the normozoospermic infertile men (median value: 0.25 ± 0.12 AU). Thus, only the difference between these two groups reached statistical significance. The data presented the statistical significance of the differences and is shown in Table 3. In the rest of the analyzed groups, there were no significant differences among subject groups in relative reactivities of LTL with PAP glycans, which reflects the amount of antennary fucose. The median values of LTL relative reactivities with seminal PAP glycans were comparable and obtained 0.19 ± 0.09 AU for control, 0.20 ± 0.15 AU for asthenozoospermic, 0.19 ± 0.16 AU for oligozoospermic, and 0.16 ± 0.1 AU for theratozoospermic group (Figure 3).

### 3.3. DC-SIGN Reactivity with Seminal Plasma Prostatic Acid Phosphatase

**Figure 4:**
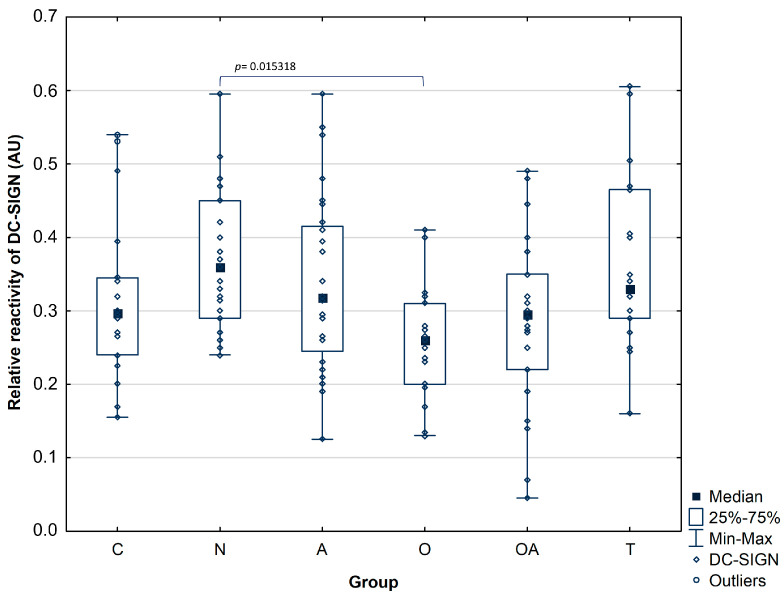
Relative reactivity of prostatic acid phosphatase glycans with DC-SIGN lectin. A *p*-value less than 0.05 was considered statistically significant. Investigated groups: C—control group, N—normozoosperic group, A—asthenozoospermic group, O—oligozoospermic group, OA—oligoasthenozoospermic group, T—teratozoospermic group.

An evaluation of seminal plasma PAP glycans binding to DC-SIGN lectin has shown that the relative reactivity of this interaction was significantly lower in the oligozoospermic group (median value: 0.26 ± 0.08 AU), with the significance level *p*^O^ = 0.015318 compared to the normozoospermic group (median value: 0.37 ± 0.1 AU, Figure 4), similar dependency was observed for relative reactivity of PAP glycans with GNL. However, no significant differences were observed among the other studied groups in the reactivity of PAP glycans with DC-SIGN, and the medians of obtained values were found as follows: control group 0.32 ± 0.11 AU, asthenozoospermic group 0.34 ± 0.12 AU, oligoasthenozoospermic group 0.29 ± 0.12 AU, and theratozoospermic group 0.37 ± 0.12 AU. The results of the analyzed parameters are summarized in Table 3.

### 3.4. Correlation Scatter Plots between DC-SIGN Reactivity and Galanthus Nivalis or Lotus Tetragonolobus Lectin Reactivity and Spearman Rank Correlation Test

**Figure 5:**
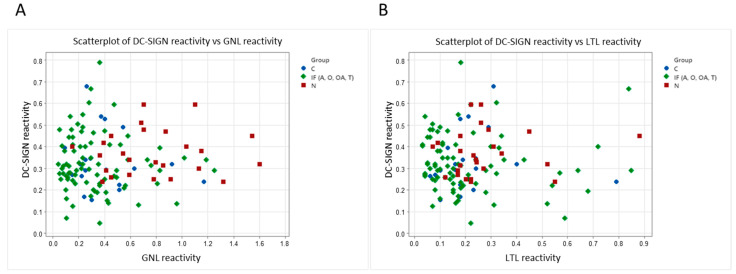
Correlation scatter plots of DC-SIGN reactivity and reactivities of PAP glycans with *Galanthus nivalis* lectin (**A**) and *Lotus tetragonolobus* lectin (**B**) for control, normozoospermic, and infertile combined (IF) group, including A—asthenozoospermic group, O—oligozoospermic group, OA—oligoasthenozoospermic group, and T—teratozoospermic subjects.

To check if the DC-SIGN reactivity is related to the mannose of fucose glycoepitopes, we analyzed the correlation of DC-SIGN vs. GNL as well as DC-SIGN vs. LTL reactivity. For this analysis, all the samples representing patients with impaired sperm patterns were combined into the infertile group (IF) and compared to the idiopathic normozoospermic group (N) and fertile controls (C). The scatter plot (Figure 5A) shows the relationship between DC-SIGN reactivity and reactivities of PAP mannose presenting glycans with *Galanthus nivalis* lectin. In contrast, Figure 5B presents the relationship between DC-SIGN reactivity and reactivity of PAP fucose containing motifs with *Lotus tetragonolobus* lectin for control, normozoospermic and combined infertile (IF) group, including asthenozoospermic (A), oligozoospermic (O), oligoasthenozoospermic (OA), and teratozoospermic (T) patients. In Figure 5A, we can observe that the data points representing the infertile combined group (IF) exhibit partially similar characteristics and are distinguished from samples representing idiopathic normozoospermic patients, presenting a separate cluster. However, we did not observe such a tendency regarding the interactions of DC-SIGN and fucose-specific lectin (Figure 5B).

Simultaneously, in the conducted statistical analysis, no Spearman’s rank correlation was found between the relative reactivity seminal plasma PAP glycans with *Galanthus nivalis* or *Lotus tetragonolobus* lectin and PAP binding to DC-SIGN, where GNL reactivity: r = –0.06, *p* = 0.54 and LTL reactivity r = −0.13, *p* = 0.22 were presented respectively, the gained results indicate that the reactivity of the investigated lectins does not unequivocally correspond to the binding of PAP to DC-SIGN.

## 4. Discussion

Seminal fluid influences different kinds of female immune responses mediated by innate and adaptive immune systems [19,20]. Immunosuppressive effects play a crucial role in mammal reproduction, as they enable sperm cells to survive in the female reproductive system, promote maternal acceptance of the embryo at implantation, and prevent allogeneic fetal rejection [19,21,22]. Human seminal plasma glycoconjugates and sperm contain high-mannose glycoforms and terminal LewisX and LewisY carbohydrate epitopes that may react distinctively with DC-SIGN expressed on myeloid antigen-presenting cells, such as macrophages and dendritic cells (DCs) [10,11,23]. C-type lectins are responsible for the identification of glycans structures in an immunological context; they can recognize “pathogen-associated molecular patterns” (PAMPs), “danger associated molecular patterns” (DAMPs) or glycosylated “self-associated molecular patterns” (SAMPs). PAMPs mainly constitute microbial products that can be detected by pattern recognition receptors (PRRs), particularly the TLRs (Toll-like receptors), nod-like receptors and dendritic cell receptors such as C-type lectins (CLRs). Pathogen interactions with Toll-like receptors can immediately trigger DCs and macrophages, resulting in the release of cytokines such as IL-12 and interferon-γ, which lead to an increase in factors that stimulate T cells. Thus, C-type lectins preset on DCs and macrophages can synergize or antagonize TLR signals, depending on the specific glycoepitopes and pathogens. Overall, the simultaneous binding of ligands to TLRs and C-type lectins might result in a tolerogenic response rather than an effector reaction [24].

It has been shown that DC-SIGN could have a dual function in the triggering of tolerance to self-antigens or recognition of pathogens [16]. DC-SIGN lectin recognizes different carbohydrate structures present on the cell surface, such as mannose-containing glycoconjugates and complex carbohydrates carrying terminal fucose in the form of LewisX and LewisY antigens. These high-complexity motifs are developmentally regulated and expressed by a variety of cells within the organism. Fucose ligands inhibit the ability of DCs to produce the inflammatory cytokines IL-6, IL-12, and thought-increasing IL-10 production, triggering an anti-inflammatory response to maintain the balance of immune homeostasis [19,25,26,27,28]. On the contrary, high-mannose-type glycans are evolutionarily conserved and, in higher mammals, may be found only in the endoplasmic reticulum during glycoprotein maturation [27,29,30]. Therefore, the exposure of high-mannose on mature glycoproteins might be considered by DC-SIGN as a sign of cell damage or pathogen invasion, directing to the initiation of mannose-mediated pro-inflammatory pathway, the occurrence of the mentioned pathway may be possible for PAP in the idiopathic normozoospermic group.

Prostatic acid phosphatase, clusterin, and galecin-3-binding protein have been recognized as endogenous glycoprotein ligands for DC-SIGN in human seminal plasma [10,31]. All of the listed major glycoproteins express multiple *N*-glycosylation sites and suggest that the presentation of LewisX and LewisY motifs on more than one *N*-glycan might be essential for DC-SIGN binding [10]. Additionally, it was indicated that these proteins feasibly complement transforming growth factor beta (TGF-β), prostaglandins, prostasomes and spermine to induce immune privilege in the male reproductive system and modulate the immune response in the female reproductive tract after insemination [31,32,33]. The mentioned seminal factors secreted by the seminal vesicle and prostate glands act as signaling agents in the female reproductive tract, where they bind to receptors on epithelial cells of the cervix and uterus, leading to activation cytokine synthesis and influencing a molecular process similar to a typical inflammatory cascade. In consequence, recruitment and activation of dendritic cells, macrophages, and granulocytes occur, which induce immunoregulatory and tissue reorganization functions that terminate with cellular changes in the endometrium to improve endometrial receptivity to facilitate successful embryo implantation and development [34].

Because PAP is found in large amounts in the seminal fluid, it is therefore believed to be an influential factor affecting the fertilization process. Former studies have not shown differences in PAP levels in groups of men with infertility problems, but many previous studies indicate that higher levels of secreted PAP are present in seminal plasma of azoospermic men, while other research described an inverse correlation between PAP level and sperm concentration in semen from oligospermic patients. Based on this report, it was postulated that PAP can be used as a marker of oligospermia [1,2]. In our study, we did not reveal statistically significant differences in the mean concentration of prostatic acid phosphatase between the control group and the infertile groups. PAP concentration was comparable in all patient groups; however, this value was slightly lower in patients with semen disorders than in the control group (Figure 1). Our results support suggestions that, in this case, potential fertility biomarkers may be identified based on altered protein modifications rather than changes in protein abundance.

When studying the modification of PAP glycans solely with plant lectins, we observed increased *Galanthus nivalis* reactivity in the samples from idiopathic normozoospermic patients. Moreover, the reactivity of PAP mannose-presenting glycans in seminal plasma was significantly higher in the asthenozoospermic group compared to the oligozoospermic and oligoasthenozoospermic groups (Figure 2). These results support our previous findings, where MALDI-MS studies regarding the *N*-glycome of seminal plasma revealed an increase in the expression of mannose-containing glycoepitopes in a group of normozoospermic men with reduced fertility [12]. This pattern was not visible in other groups of infertile men, where a decrease in available mannose residues was found. In that study, high mannose and hybrid-type glycans comprised 15–28% of the total *N*-glycome; their content was the highest in normozoospermic men (28%) and lowest in the oligozoospermic samples (15%), which is consistent with our current research, this may indicate that PAP is the main glycoprotein bearing unique high-mannose and hybrid type oligosaccharides in seminal fluid. Another confirmation of this suggestion may be our mass spectrometry identification of seminal plasma proteins isolated by GNL affinity chromatography, where PAP was observed as the major *Galanthus nivalis* lectin-bound glycoprotein [12,35]. Investigating LTL reactivity with PAP, we have found no differences in the relative reactivities of PAP glycans with *Lotus tetragonolobus* lectin among individuals with confirmed fertility and groups of patients with abnormal semen parameters, apart from lower amounts of antennary fucose in seminal plasma of oligoasthenozoospermic group in comparison to the normozoospermic group. Similar results were obtained in our previous investigation by applying less specific *Aleuria aurantia* lectin [36]. In conclusion, the results proved herein indicate that the PAP fucosylation pattern does not show intense variability among the studied groups contrary to the mannosylation profile.

As plant lectins serve as a model system to evaluate the interactions possibly present in vivo, we finally examined the reactivity of seminal plasma PAP glycans with DC-SIGN. Similarly to the reactivity of GNL with PAP glycans, we observed a significantly higher relative reactivity in the normozoospermic group than the oligozoospermic group; however, no significant differences were noted between the other studied groups (Figure 4). Prostatic acid phosphatase presents the above-discussed types of oligosaccharide structures, potential DC-SIGN ligands. The analysis of PAP reactivity with DC-SIGN confirms that this glycoprotein is a lectin ligand but does not allow us to assess which glycoepitopes is involved. A comparison of DC-SIGN reactivity with the interaction of plant lectins that distinguish both glycoepitopes allows us to suggest that in the studied material, the content of high-mannose oligosaccharides is a feature able to distinguish natural interactions among patients with different reproductive potential, as shown in scatter plots (Figure 5), in which a tendency to form separate clusters are observed for patients with idiopathic normozoospermia and those with infertility related to abnormal semen parameters. Such clustering was not observed when comparing the interactions of DC-SIGN and fucose-specific lectin. However, Spearman’s rank correlation analysis did not confirm the correlation between the relative reactivity of seminal plasma PAP glycans with *Galanthus nivalis* or *Lotus tetragonolobus* and the binding of PAP to DC-SIGN. However, it is also possible that the binding of the glycans to CRD domains of DC-SIGN can depend on subtle differences in the distribution and composition of monosaccharide subunits and on fine distinctions in the spatial arrangements of these molecules. Additionally, oligomerization of the four CRD domains of DC-SIGN may alter the affinity and specificity properties of this binding [16,37]. In future studies, it would be worth considering the use of Ig fusion protein, DC-SIGN-Fc chimera protein, which has the antigen binding site on each arm of the IgG chimera and therefore has the bivalent nature, which might affect the binding efficiency of the molecule [16,38].

We suggest that the high concentration of prostatic acid phosphatase and its abundance of mannosylated and highly fucosylated glycan functional groups in human semen suggest that PAP might be one of the glycoproteins involved in the modulation of the immune response. In addition, PAP provides a shift in the function of DCs into a regulatory profile that promotes a tolerogenic response to male antigens in the female reproductive system, thereby contributing to the protection of human gametes and successful pregnancy.

## Figures and Tables

**Table 1:** The characteristics of the samples comprising the mean values ± SD (standard deviation), median and range.

Group	Subject Age [Years]			Sperm Count [×10^6^/mL]			Sperm Motility [%]		
	Mean ± SD	Median	Range	Mean ± SD	Median	Range	Mean ± SD	Median	Range
C (*n* = 20)	31 ± 5	32	22–39	56.3 ± 35.0	52.7	20.4–99.2	55 ± 11	56	36–71
N (*n* = 25)	34 ± 4	34	28–42	61.8 ± 22.0	61.5	23.0–109.7	51 ± 10	52	33–67
A (*n* = 28)	34 ± 5	32	29–47	35.2 ± 18.9	28.6	19.0–94.7	25 ± 7	26	4–30
O (*n* = 20)	32 ± 4	32	27–40	7.9 ± 2.0	8.2	3.0–11.2	46 ± 11	41	33–67
OA (*n* = 23)	33 ± 6	33	24–49	6.1 ± 4.0	6.4	0.3–10.8	18 ± 9	18	1–29
T (*n* = 19)	37 ± 6	37	33–41	62.0 ± 19.0	68.0	40.4–77.6	40 ± 23	48	14–58

Investigated groups: C—control group, N—normozoosperic group, A—asthenozoospermic group, O—oligozoospermic group, OA—oligoasthenozoospermic group, T—teratozoospermic group.

**Table 2:** The values of PAP concentration in seminal plasma according to the control group and patient groups.

**PAP** **concentration** **(mg/mL)**		**Group**					
		**C**	**N**	**A**	**O**	**OA**	**T**
		***n*** **=** **20**	***n*** **=** **25**	***n*** **=** **28**	***n*** **=** **20**	***n*** **=** **23**	***n*** **=** **19**
	Mean ± SD	0.83 ± 0.57	0.71 ± 0.40	0.66 ± 0.46	0.61 ± 0.35	0.61 ± 0.31	0.65 ± 0.44
	Median	0.69	0.73	0.49	0.52	0.54	0.49
	Range	0.27–1.29	0.46–0.89	0.34–0.75	0.39–0.83	0.38–0.79	0.30–0.87

PAP—prostatic acid phosphatase concentration (mg/mL). A *p*-value less than 0.05 was considered statistically significant.

**Table 3:** Lectin reactivity values of seminal plasma PAP glycans according to the control group and patient groups.

Lectins Reactivity (AU)		Group					
		C	N	A	O	OA	T
		*n* = 20	*n* = 25	*n* = 28	*n* = 20	*n* = 23	*n* = 19
GNL reactivity	Mean ± SD	0.41 ± 0.27	0.78 ± 0.38	0.48 ± 0.30	0.26 ± 0.23	0.21 ± 0.12	0.25 ± 0.17
			*p*^OA^ = 0.000001 *p*^O^ = 0.000002 *p*^T^ = 0.000013 *p*^C^ = 0.043308	*p*^OA^ = 0.007676 *p*^O^ = 0.036319			
	Median	0.30	0.70	0.46	0.19	0.22	0.22
	Range	0.24–0.51	0.45–1.03	0.26–0.58	0.1–0.36	0.1–0.34	0.15–0.31
LTL reactivity	Mean ± SD	0.19 ± 0.09	0.25 ± 0.12	0.20 ± 0.15	0.19 ± 0.16	0.17 ± 0.15	0.16 ± 0.1
			*p*^OA^ = 0.046826				
	Median	0.18	0.23	0.17	0.15	0.14	0.15
	Range	0.13–0.24	0.18–0.28	0.10–0.28	0.08–0.26	0.09–0.19	0.08–0.22
DC-SIGN reactivity	Mean ± SD	0.32 ± 0.11	0.37 ± 0.1	0.34 ± 0.12	0.26 ± 0.08	0.29 ± 0.12	0.37 ± 0.12
			*p*^O^ = 0.015318				
	Median	0.30	0.36	0.32	0.26	0.30	0.33
	Range	0.24–0.35	0.29–0.45	0.25–0.42	0.20–0.31	0.22–0.35	0.29–0.47

GNL reactivity—reactivity of PAP glycans with *Galanthus nivalis* lectin (AU); LTL reactivity—reactivity of PAP glycans with *Lotus tetragonolobus* lectin (AU); DC-SIGN reactivity—reactivity of PAP glycans with DC-SIGN (AU). The *p*-values less than 0.05 were considered statistically significant. Lectins reactivity is shown in arbitrary units (AU) as mean values ± standard deviation (SD).

## Data Availability

The data underlying this article will be shared on reasonable request to the corresponding author.
